# Aggressive vocal expressions—an investigation of their underlying neural network

**DOI:** 10.3389/fnbeh.2015.00121

**Published:** 2015-05-11

**Authors:** Hannah S. Klaas, Sascha Frühholz, Didier Grandjean

**Affiliations:** ^1^Neuroscience of Emotion and Affective Dynamics Laboratory (NEAD), Department of Psychology, University of GenevaGeneva, Switzerland; ^2^Swiss Center for Affective Sciences, University of GenevaGeneva, Switzerland

**Keywords:** emotion, voice, vocal production, neural network, fMRI

## Abstract

Recent neural network models for the production of primate vocalizations are largely based on research in nonhuman primates. These models seem yet not fully capable of explaining the neural network dynamics especially underlying different types of human vocalizations. Unlike animal vocalizations, human affective vocalizations might involve higher levels of vocal control and monitoring demands, especially in case of more complex vocal expressions of emotions superimposed on speech. Here we therefore investigated the functional cortico-subcortical network underlying different types (evoked vs. repetition) of producing human affective vocalizations in terms of affective prosody, especially examining the aggressive tone of a voice while producing meaningless speech-like utterances. Functional magnetic resonance imaging revealed, first, that bilateral auditory cortices showed a close functional interconnectivity during affective vocalizations pointing to a bilateral exchange of relevant acoustic information of produced vocalizations. Second, *bilateral* motor cortices (MC) that directly control vocal motor behavior showed functional connectivity to the *right* inferior frontal gyrus (IFG) and the *right* superior temporal gyrus (STG). Thus, vocal motor behavior during affective vocalizations seems to be controlled by a right lateralized network that provides vocal monitoring (IFG), probably based on auditory feedback processing (STG). Third, the basal ganglia (BG) showed both positive and negative modulatory connectivity with several frontal (ACC, IFG) and temporal brain regions (STG). Finally, the repetition of affective prosody compared to evoked vocalizations revealed a more extended neural network probably based on higher control and vocal monitoring demands. Taken together, the functional brain network underlying human affective vocalizations revealed several features that have been so far neglected in models of primate vocalizations.

## Introduction

Affective vocalizations play an important role in human and animal communication. The production of such affective vocalizations is based on a neural cortico-subcortical network summarized in several recent models (Lauterbach et al., 2013; Ackermann et al., 2014). These models take strong reference to studies in nonhuman primates and in nonhuman mammals (Hage and Jürgens, 2006). They point to two hierarchical organized pathways involved in voluntary “vocal control and patterning” and in rather involuntary “vocal initiation” (Jürgens, 2009; Lauterbach et al., 2013). For “vocal initiation”, the cingulo-limbic-brainstem pathway includes regions like the reticular formation, the periaqueductal gray (PAG), and the anterior cingulate cortex (ACC). These regions are supposed to serve vocal pattern generation, gating of the intensity of a vocal response, and (involuntary) initiation especially of emotional vocalizations, respectively. The motor cortical vocal control pathway (“vocal control and patterning”) involves several feedback loops including the (pre-)motor cortex, the basal ganglia (BG), the cerebellum (Cbll), the thalamus, and pontine regions. These regions serve to optimize and monitor rather controlled and voluntary vocalizations and vocal expression of affect.

Though these recent models of primate vocalizations provide a detailed description of the neural network underlying the production of vocalizations and vocal expression of emotions, there are some open questions remaining. These questions especially concern the neural network underlying more complex human affective vocalizations (e.g., affective prosody) beyond the network similarities for human and nonhuman primates. We specifically choose to investigate the neural network underlying the production of “hot” anger superimposed on speech-like utterances (i.e., affective prosody). Affective prosody is a human-specific expression of vocal emotions, wherein hot and aggressive anger is a vocalization that can be reliably analyzed in terms of acoustical parameters (Banse and Scherer, 1996; Patel et al., 2011). Aggressive anger also considerably drives brain network responses and dynamics (Frühholz and Grandjean, 2012; Frühholz et al., 2015), and it also includes considerable body physiological changes (Aue et al., 2011; Frühholz et al., 2014a). Investigating the neural network underlying the production of hot and aggressive anger enabled us to address some of the remaining questions mentioned above. First, some open questions concern the specific functional role of certain brain areas in this network. Second, these open questions also concern some brain regions, which are not included in previous network models, but which seem to be central to the production especially of human affective vocalizations. A third and final question is related to the laterality of the network that underlies different types of affective vocalization productions (Ross and Monnot, 2008).

Concerning the first question of the specific functional and network role of certain brain areas, two regions seem to be specifically important. The ACC, for example, is supposed to be a central structure in the production of nonhuman affective vocalizations (Jürgens, 2009). It was surprisingly not proposed, however, to be relevant for human affective vocalizations with volitional control (Jürgens, 2002; Ackermann, 2008). Only recently, the ACC has been included in neural models of vocalizations. It was proposed to be a cingulate vocalization area that releases stereotyped motor patterns of affective-vocal displays (Ackermann et al., 2014) probably based on its connections to the striatum (Ongür and Price, 2000) and to the PAG (Jürgens, 2009; Hage et al., 2013). There are, however, several recent studies pointing to a central role of the ACC in human affective prosody production, even when volitional control is involved. For example, the ACC has recently been found during the inhibition and voluntary production of laughter (Wattendorf et al., 2013). ACC activity has also been found in relation to pitch modulations and has been connected negatively to pitch range (Barrett et al., 2004). Furthermore, this brain region is implicated in a system of emotional control and of affective autonomic response generation (Critchley, 2009).

Besides this discussion of the functional role of the ACC, the functional role and the connectivity of the BG is also under debate. Recent neuroimaging studies (Arnold et al., 2013; Pichon and Kell, 2013; Frühholz et al., 2014a) point to an involvement of the BG in human affective voice production and patterning of (learned) vocalizations (Jürgens, 2009). This seems to be based on a functional connectivity of the BG to the amygdala, hippocampus, and the motor cortices (MC) during the production of vocal emotions (Pichon and Kell, 2013). This function of patterning of the BG was also recently discussed as their specific role in the temporal sequencing of vocal utterances (Kotz and Schwartze, 2010). However, beyond this positive role of the BG by preparing and sequencing vocal output, the BG might also have more regulatory and partly inhibitory functions during vocal output (Gale and Perkel, 2010; Tressler et al., 2011), which is far less understood especially in humans. Thus, the first major aim of the present study was to determine the functional role and the functional connectivity of the BG and the ACC in the neural network underlying human affective vocalizations.

Concerning the second major question about brain regions, which have been rather neglected so far in neural network models, there are again two regions that might be central to such a network, and which should be strongly coupled in terms of their functional roles. First, recent findings suggest the primate inferior frontal gyrus (IFG) being involved in the planning and initiation (Hage and Nieder, 2013) of primate expressions with a higher level of cognitive control (Hage et al., 2013). The latter might be especially the case with human affectively intonated speech. The IFG might have a specific role in vocal monitoring during the production of vocal affect (Frühholz et al., 2014b). Second, the role of the IFG might be tightly linked to activity in auditory cortical regions in superior temporal gyrus (STG) during more complex vocalizations (Frühholz and Grandjean, 2013; Pichon and Kell, 2013; Frühholz et al., 2014a). In songbirds, for example, learning songs from a model includes activations of the auditory cortex in the STG during song processing and production (Mooney, 2004). This STG activity could be based on a feedback-loop during the comparison of the own song production to a tutor produced song (Mandelblat-Cerf et al., 2014) or based on the memory retrieval of learned sounds (Miller-Sims and Bottjer, 2014). This together is in accordance with results from human neuroimaging studies. STG activity in humans is likely to provide auditory feedback-monitoring loops as well as short-term sound memory in the production of the affectively intonated vocal utterances (Pichon and Kell, 2013; Frühholz et al., 2014a). Yet the close connectivity of the STG and the IFG and its relationship to cortical and subcortical motor structures, which directly control the vocal output, is not fully understood yet. Therefore, we hypothesize a central role of the STG in connection with the IFG during the motor production of human affectively intonated utterances.

The third and final question concerned the role of a network lateralization during affective prosody production, which so far produced inconsistent results. Lesion studies point to a dominant role of the right hemisphere for controlling the paralinguistic dimension of human vocalizations in terms of prosody (Ross and Monnot, 2008). Neuroimaging studies however predominantly found bilateral activations (Laukka et al., 2011; Pichon and Kell, 2013; Frühholz et al., 2014a) and a rather bilateral network underlying the production of affective prosody (Arnold et al., 2013; Pichon and Kell, 2013). Thus, investigating the functional connectivity between regions that are involved in the production of affective prosody could provide insights into the organization and relevance of the left and/or right brain network that is also relevant for different types of vocal production. Concerning the latter, there is evidence from patient studies that networks differ between different types of production, especially between the repetition (i.e., listening to and imitating another speaker) and the evoked production of affective prosody (i.e., individual expression of prosody) (Heilman et al., 2004; Ross and Monnot, 2008). We thus included both production types in this study. Though both types of vocal production of affective prosody have to be initiated volitionally, we expected to identify a more extended functional network during the repetition than during the evoked production of affective prosody. This more extended network might reflect higher level of cognitive control and monitoring demands over the acoustic structure of the prosody during the repetition of affective prosody. Therefore, we specifically expected a stronger connectivity between the IFG and temporal STG regions responsible for adjustment and monitoring of acoustical such as spectral and temporal features for the repetition of affective prosody.

Taken together the present study tested several new hypotheses about the functional network role of specific brain regions. First, unlike the common view that the ACC mainly involuntarily releases vocal patterns (Jürgens, 2009; Ackermann et al., 2014) we hypothesized that the ACC is also involved in more controlled human affective vocalizations. This was hypothesized especially based on the connectivity of the ACC to other important cortical regions of the vocalizations network. In terms of this perspective, the ACC might monitor errors in terms of vocal performance (Carter et al., 1998) and might voluntarily regulate the intensity of vocalizations based on the bodily arousal (Rudebeck et al., 2014). Second, besides the assumed role of the BG in vocal patterning, we hypothesized that the BG are specifically involved in more regulatory and inhibitory neural network mechanisms that shape vocal productions (Lu et al., 2010; Ravizza et al., 2011; Chang and Zhu, 2013; Péron et al., 2013). Third, we hypothesized a close IFG-STG connectivity during controlled vocalizations that supports voluntary vocal monitoring based on acoustic feedback processing (Frühholz et al., 2014b). Finally, in relation to the different types of vocal productions we expected to find an extended and partly right lateralized neural network during the repetition of affective prosody. This was hypothesized based on data from patient studies (Ross and Monnot, 2008), and we expected that this network directly influences cortically controlled vocal motor behavior during affective vocalizations.

## Materials and Methods

### Participants

Fifteen healthy, native French-speaking and right-handed volunteers participated in the experiment, but two participants had to be excluded due to insufficient vocal performance (Frühholz et al., 2014a). The final sample thus consisted of 13 healthy, native French-speaking and right-handed volunteers that participated in this study (seven female, mean age 23.85 years, SD 3.69, age range 19–32 years). They had normal or corrected-to-normal vision and normal hearing abilities, and no history of psychiatric or neurologic disorders. Participants gave informed and written consent for their participation in the experiment. The study was approved by the local ethics committee in accordance with ethical and data security guidelines of the University of Geneva.

### Stimulus Material and Task Procedure

During the experiment, participants had to express neutral and angry prosody on five-letter pseudowords consisting of consonant-vowel combinations (i.e., “belam”, “lagod”, “minad”, “namil”). The same four pseudowords were also chosen from a sample of different pseudowords previously spoken by two male and two female actors in a neutral and angry tone before the experiment. A total of 32 pseudowords (2 male actors/2 female actors × 4 pseudowords × 2 emotions) were selected and then normalized for the mean energy across all stimuli (Frühholz et al., 2014a). The experiment consisted of four experimental blocks represented by two repeated production blocks and two evoked production blocks. Across the experiment repetition and evoked production blocks alternated. The block sequence was counterbalanced across participants. The 38 trials of each block consisted of 32 trials with prosody productions and six null events during which no stimulus appeared and participants were told to rest. The order of the trials was randomized for each participant.

In repetition blocks, participants were asked to repeat the prosodic intonations, which they immediately heard spoken beforehand by the actor recordings. The evoked production blocks included a freely acted production of prosody with no constraint of imitating or repeating a previously heard prosodic style of an actor. In both tasks, the pseudoword was first presented on a gray screen for 800 ms starting 250 ms after the last volume acquisition. It was presented either in lowercase letters (indicating neutral prosody production) or in uppercase letters (indicating angry prosody production). The word was presented together with the voice of the actors during the repetition task followed by a visual black cross during one volume acquisition (TA = 1580 ms, see below). After the volume acquisition, the black cross turned into a white cross, indicating that participants should produce the prosody asked for. The white cross remained on the screen for 1580 ms, after which the cross turned black again during the next volume acquisition. We used an fMRI-compatible Sennheiser optical microphone (16 bit, 44.1 kHz) and a digital voice recorder to register participants’ prosody productions in the silent gap during volume acquisition.

### Functional Localizer Scanning

The experiment included two localizer scans. First, we determined human voice-sensitive regions in the bilateral STG by using 8 s sound clips taken from an existing database^1^ (Belin et al., 2000). The sounds clips consisted of 20 sequences of animal or environmental sounds and 20 sequences of nonemotional human voices. Each sound clip was presented once. The scanning sequence also contained 20 8 s silent events. Participants listened passively to the stimuli.

Second, to be able to reveal sensorimotor regions showing activations especially due to mouth movement underlying the execution of prosody productions, we conducted a movement localizer scan. The movement localizer consisted of eight resting blocks and eight movement blocks. In each block, the same word appeared 10 times, alternating with a cross every 1 s. In movement blocks, participants were instructed to form the word with their lips as soon as it appeared on the screen. In resting blocks, they were instructed to restrain from any lip movement. Movement and resting blocks were separated by 5 s gaps. For the mouth movement localizer we used the words of the main experiment, and each word was used in two movement blocks and in two resting blocks.

### Image Acquisition and Image Processing

Functional imaging data were acquired on a 3T Siemens Trio System (Siemens, Erlangen, Germany) using a T2*-weighted gradient echo planar imaging sequence (TR = 3290 ms, TA = 1580 ms, TE = 30 ms, FA = 90°, 28 slices, slice thickness 4 mm, distance factor = 20%, 64 matrix (3 × 3 mm)). The use of a sparse temporal acquisition protocol for the main experiment allowed to present auditory stimuli in the silent gap between volume acquisitions and to record the prosody productions of the participants. A high-resolution magnetization prepared rapid acquisition gradient echo T1-weighted sequence (1 mm slices, TR = 1900 ms, TE = 2.27 ms, TI = 900 ms, FoV 296 mm, in-plane 1 × 1 mm) was obtained in sagittal orientation to obtain structural brain images from each participant.

We used the Statistical Parametric Mapping software SPM8 (Welcome Department of Cognitive Neurology, London, UK) to preprocess images from the main experiment and from both localizer scans. Functional images were realigned and coregistered to the anatomical image. We ensured that head movements of the participants were less than half of the voxel size used for image acquisition. Segmentation of the anatomical image revealed warping parameters that were used to normalize the functional images to the Montreal Neurological Institute (MNI) stereotactic template brain. Functional images were resampled to a 2 mm^3^ voxel size and spatially smoothed using an isotropic Gaussian kernel of 8 mm^3^ FWHM.

### Functional Connectivity Analysis

Our previous analysis revealed a distributed pattern of activations in a fronto-temporal and subcortical network of regions underlying the expressions of vocal anger. This activity mainly included frontal activity in the left (MNI xyz [−38 26 2]) and right IFG ([52 24 −6]), the ACC ([−2 16 34]), subcortical activity in the left Putamen (Put, [−25 5 14]) and right caudate nucleus (Cd, [10 2 6]). Activity was also found in the left temporal cortex, such as mSTG ([−54 −10 8]) and pSTG ([−52 −24 8]) and hippocampus (HC, [−28 −42 −2]), as well as activity in right mSTG ([54 −20 4]). These seed regions were chosen because of several open questions concerning the functional role of some regions (i.e., ACC, BG) in the neural vocalizations network as outlined in the introduction. They were also chosen because of their proposed importance in the cortico-subcortical network underlying human vocalizations (Arnold et al., 2013; Pichon and Kell, 2013; Frühholz et al., 2014a, b). Especially, the IFG is assumed to provide vocal monitoring during affective vocalizations (Frühholz et al., 2014b), probably based on auditory-feedback processing in the STG (Pichon and Kell, 2013; Frühholz et al., 2014a). Thus we assumed that both regions are critical components of a neural vocalizations network, which have been rather neglected so far (Ackermann et al., 2014). Furthermore, the functional role and connectivity of the HC was especially tested for the type of evoked vocalizations, because it was previously shown to have a specific functional role for this type of vocalization in terms of the retrieval of long-term stored vocal scripts (Frühholz et al., 2014a).

These seed regions were subjected to a psycho-physiological interaction (PPI) analysis (Friston et al., 1997). The PPI analysis aims to model activity in other brain regions based on the time course of the functional activity in a seed region. A seed and a target region are assumed to be functionally connected if brain activity in the target region can be explained based on a model. The model results from multiplying the time course activity in the seed region with a binary comparison of task conditions (“1” and “−1”, see below). This time course multiplied by the comparison of task conditions represents the interaction between the physiological and the psychological variable, respectively. We extracted the time course of activation in the seed regions using a 3 mm radius sphere around group-level peak activation applied to each participant.

The PPI analysis was set up as a general linear model for the production of angry compared with neutral prosody separately for each task and for each seed region including three regressors for each analysis. The first regressor included the extracted and deconvolved time course of functional activity in a seed region (the physiological variable). The second regressor represented the comparison of angry and neutral productions during the task (the psychological variable), that is, we created a time course regressor for the task including as many sampling points as for the physiological variable. The values in this regressor were set to “1” for trials including angry productions and to “−1” for trials including neutral productions. Only trials were included in the PPI analysis where participants validly produced the target emotion corresponding to 84% of the angry trials and 81% of the neutral trials (Frühholz et al., 2014a). The third regressor included the interaction between the first two regressors as represented by a point-by-point multiplication of the time course for the physiological variable and the time course for the psychological variable. The last regressor was the only regressor of interest, whereas the psychological variable and the deconvolved time course served as regressors of no interest in each PPI analysis. The inclusion of the first two regressors ensures that the resulting functional activation is solely determined by the *interaction* between the physiological variable and the psychological variable.

For each seed region separately, the single-subject PPI data for the repetition task and for the evoked task were entered into a second-level random effects analysis. On the second level of the analysis contrasts were computed of positive and negative functional connectivity, which was common to both tasks (repetition and evoked prosody production), and functional connectivity, which was higher for one compared with the other task. All contrasts were thresholded at *p* < 0.001 and a cluster extent of *k* = 34. This combined voxel and cluster threshold corresponds to *p* < 0.05 corrected at the cluster level. This was determined by the 3DClustSim algorithm implemented in the AFNI software^2^ using the estimated smoothness of the data across all contrasts computed. Across all contrasts this procedure resulted in a maximum *k* = 34, and this was set as cluster threshold for all contrasts.

## Results

**Figure 1:**
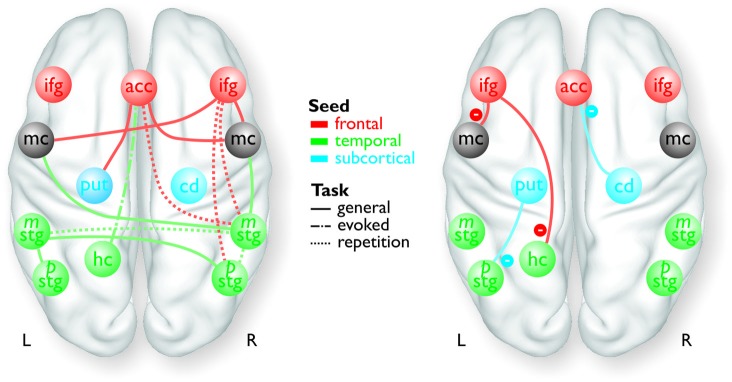
**Overview of the frontal (red), temporal (green), and subcortical seed regions (blue) included in the PPI analyses, except for the bilateral MC and the right pSTG, which only resulted as target regions in the analyses**. The connections between the regions represent a summary of the functional pathways with positive functional connectivity (left panel) and with negative functional connectivity (right panel) as determined by the PPI analyses. The color of the pathways is according to the color of the seed region.

The PPI analysis revealed a widespread functional neural network underlying the expression of affective prosody of “hot” and aggressive anger (Figure 1; Table 1). All functional connections survived a threshold of *p* < 0.05 corrected at the cluster level. Here, we were specifically interested in functional connections between the seed regions of the PPI analysis and all brain regions located in the voice-sensitive STG. We were additionally interested in functional connectivity to the frontal areas of the MC as determined by the mouth movement localizer scan (see Table 1 for a full list of functional connections). The latter regions are part of the MC that directly controls vocal tract behavior during vocalizations.

**Table 1:** **Results of the functional connectivity analyses for seed regions in (A) the frontal cortex (i.e., left and right IFG, ACC), (B) subcortical regions (i.e., left Put, right Cd), and (C) the medial (i.e., left HC) and lateral temporal cortex (i.e., mSTG)**.

Region	Cluster size	*t* value	MNI		
			*x*	*y*	*z*
**(A) ACC: general**
L putamen	93	5.09	−12	−2	8
R precentral gyrus	41	4.89	52	−12	36
**ACC: repetition > evoked**
R superior temporal gyrus	108	4.64	60	−18	6
R superior temporal gyrus		4.03	58	−34	12
**Left IFG: general (−)**
L superior parietal lobule	321	7.29	−38	−78	38
L superior occipital gyrus		4.93	−22	−80	40
L middle occipital gyrus	76	5.31	−44	−74	28
L angular gyrus		3.61	−36	−66	30
L middle frontal gyrus	51	5.08	−30	16	50
L precentral gyrus	81	4.78	−52	6	32
L hippocampus	38	4.54	−32	−30	−14
**Right IFG: general**
L precentral gyrus	103	5.76	−56	−6	24
R precentral gyrus	101	4.44	48	−12	38
**Right IFG: repetition > evoked**
R superior temporal gyrus	364	5.83	62	−12	4
R superior temporal gyrus		4.58	60	−36	8
**(B) Left Puts: general (−)**
L superior temporal gyrus	105	4.91	−48	−36	8
L middle occipital gyrus	94	4.55	−38	−76	32
**Right Cd: general (−)**
L cingulate gyrus	69	5.52	−6	34	28
R inferior parietal lobule	180	5.45	52	−30	52
R middle frontal gyrus	67	5.39	12	0	44
L superior parietal lobule	45	5.23	−16	−54	66
R middle frontal gyrus	87	5.21	26	26	44
R middle frontal gyrus	99	4.79	40	22	34
L paracentral lobule	61	4.75	−2	−42	66
R superior frontal gyrus	184	4.50	30	−8	66
R precentral gyrus		4.06	32	−10	60
**(C) Left mSTG: general**
L superior temporal gyrus	34	4.82	−64	−30	2
**Right mSTG: general**
R precentral gyrus	63	4.74	46	−12	40
L precentral gyrus	45	4.24	−58	−10	44
**Right mSTG: repetition > evoked**
R superior temporal gyrus	285	6.70	62	−34	14
L superior temporal gyrus	39	4.23	−52	−18	0
L superior temporal sulcus		3.75	−52	−14	−6
**Left HC: evoked > repetition**
L cingulate gyrus	36	4.77	−14	8	44

*Functional connections were obtained independently of the task (general), or were significantly increased for the repetition task (repetition > evoked) or for the evoked task (evoked > repetition). Negative functional connections are indicated by a minus in brackets (−)*.

**Figure 2:**
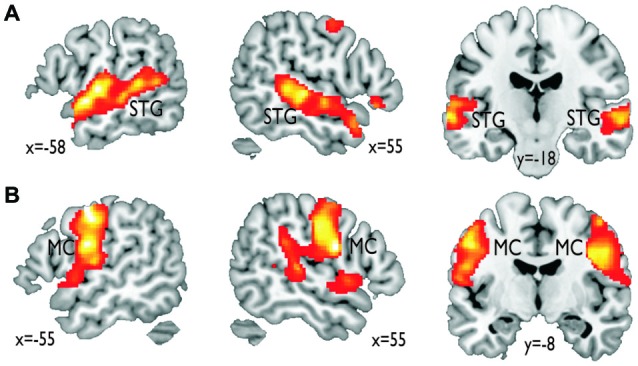
**Functional activations resulting from the functional localizer scans. (A)** Vocal compared to nonvocal sounds during the voice localizer scan revealed an extended bilateral activity in the superior temporal gyrus (STG). **(B)** Mouth movement compared with the baseline revealed extended activity in bilateral inferior motor cortex (MC).

In terms of localizing cortical voice-sensitive regions, vocal compared to nonvocal sounds during the voice localizer scan revealed extended activity in bilateral STG (Figure 2A), which is in line with many recent studies using the same localizer scan (Belin et al., 2000; Frühholz et al., 2012) and with a meta-analysis on affective voice sensitivity in STG (Frühholz and Grandjean, 2013). In terms of cortical vocal motor areas, the mouth movement localizer scan revealed activity, which was located mainly in the lateral inferior MC (Figure 2B), which also has been previously reported (Lotze et al., 2000; Meier et al., 2008). Functional activations for the two localizer scans also survived a threshold of *p* < 0.05 corrected at the cluster level.

**Figure 3:**
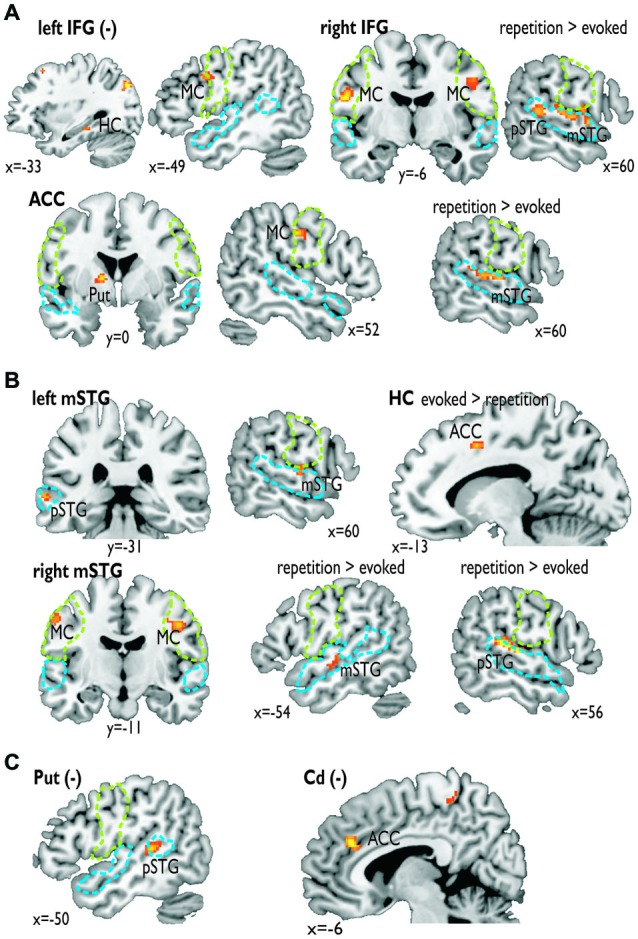
**Functional connectivity for seed regions in (A) the frontal cortex (i.e., left and right IFG, ACC), (B) the medial (i.e., left HC) and lateral temporal cortex (i.e., mSTG), and (C) subcortical regions (i.e., left Put, right Cd).** The seed regions are indicated by the bold label. A minus in brackets (−) indicates a negative functional connectivity. Some functional connections were significantly greater during the repetition task (repetition > evoked) or the evoked task (evoked > repetition). The green dashed line denotes the area of the motor cortex as determined by the mouth movement localizer scan; the blue dashed line denotes the voice sensitive temporal cortex as determined by the voice localizer scan.

For the functional connectivity analysis we revealed a differential connectivity pattern for the different frontal seed regions. While the left IFG revealed a negative connectivity with the HC and especially with the left inferior MC, the right IFG and the ACC revealed positive connections to the bilateral and right inferior MC, respectively (Figure 3A). All MC activations were located in the functional area as determined by the mouth movement localizer scan. Thus, the connection of right IFG to bilateral MC indicate that the cortical vocal motor regions are positively linked to right IFG regions, but negatively to left IFG regions. The right IFG and ACC also revealed positive connections to right STG regions located in the voice sensitive cortex as determined by the functional voice localizer scan. The latter connections and especially the connection between IFG and STC were increased during the repetition task, which was expected by one of our hypotheses. The ACC also revealed a positive connectivity to the left Put and negative connectivity to the right Cd.

Similar to frontal seed regions, seed regions in the temporal cortex revealed a differential pattern of connectivity. The right mSTG as a seed region also revealed positive connections to bilateral MC (Figure 3B). This again indicates that cortical vocal motor regions are predominantly linked to right, but not to left auditory regions. Besides this connectivity to frontal brain regions the right mSTG also showed connectivity to bilateral temporal regions in the voice-sensitive cortex, namely to ipsilateral pSTG and to contralateral mSTG. As hypothesized, the latter connections were again significantly higher especially during the repetition task. The left mSTG revealed a similar bilateral connectivity to temporal voice-sensitive regions of the pSTG, but these connections were not significantly increased during one of the tasks.

Besides the cortical frontal and temporal seed regions, we also investigated the functional connectivity patterns of two subcortical seed regions located in the BG (Figure 3C). The subcortical seed regions in left Put and in right Cd only revealed a negative functional connectivity to left pSTG and to the ACC, respectively. This highlights the hypothesized regulatory and partly inhibitory role of the BG during the shaping of human affective vocalizations.

## Discussion

Our study aimed at identifying the functional neural network involved in affective prosody production especially for aggressive vocalizations of “hot anger”. We also aimed at identifying the neural network for different levels of vocal control and monitoring demands according to different production types of vocalizations. The functional connectivity data include several important findings: first, the right hemisphere plays a dominant role in affective prosody production, namely right frontal and right auditory MC that regulate vocal motor behavior of vocalizations. Second, both the IFG and the STG have been largely neglected in recent neural models of vocalizations, but our data point to their critical role during vocalizations, probably related to vocal monitoring and vocal feedback processing. We especially revealed a more extended functional fronto-temporal neural network for the repetition relative to the evoked production condition. The connectivity of the right IFG with the ipsilateral STG was increased during the repetition condition pointing to increased monitoring demands during the imitation of previously heard vocalizations. Third, our data expand neural vocalization models, by also pointing to the central role of the ACC and the BG in this network.

The distinct functional roles of ACC and the BG were the concern of our first question outlined in the introduction. The ACC has been suggested to figure as a cingulate vocalization area that releases stereotyped motor patterns of affective-vocal displays (Ackermann et al., 2014). This release is probably based on the ACC connection to the striatum (Ongür and Price, 2000), which generally underlies overlearned behavioral patterns (Graybiel, 2005). Our data confirmed this functional connection of the ACC with the BG, additional to a functional connection to the right MC, thus supporting the view of the ACC as a neural node to release patterns of affective vocal displays and map them to the MC. We also found a connectivity of the ACC with the HC during the evoked production of vocalizations. It is likely that the HC includes long-term stored scripts of learned prosodic patterns rather than preprogrammed scripts. These scripts might be retrieved and released during the evoked production of vocalizations, whereas the ACC connections to the right STG might release patterns of vocalizations stored in short-term memory (Frühholz et al., 2014a). Besides this role of the ACC in releasing vocal patterns, the ACC has been recently also found to control the bodily arousal level (Rudebeck et al., 2014) that accompanies aggressive vocalizations (Frühholz et al., 2014a). The ACC thus might regulate the arousal and intensity level during affective vocalizations, probably by regulating the intensity level of vocal motor responses through its connectivity with the BG and the MC. Finally, the ACC is also assumed to generally monitor errors in overt performances (Carter et al., 1998). It might thus be involved in detecting vocalizations errors, given its connection with the right STG especially during the repetition condition. Similar connections to the right STG were also found for the right IFG during the repetition condition. The STG and the IFG together support a combined error detection (ACC) and monitoring (IFG) of repeated vocalizations feedback to and analyzed by the right STG.

Besides the ACC, our study also aimed to determine the functional role and the connectivity of the BG during affective vocalizations. The BG have been proposed to be involved in the generation and suprasegmental sequencing of temporal vocal patterns (Kotz and Schwartze, 2010). These temporal patterns of central acoustic features of affective vocalizations are important for affective prosody (Pell and Kotz, 2011; Frühholz et al., 2014c). The correct production of these features helps listeners to categorize these vocalizations (Banse and Scherer, 1996). This temporal sequencing in the BG might be directly coupled with the release of vocal patterns by the ACC.

Beyond this potential role of the BG in temporal sequencing of vocalizations, we found two negative functional connections of the left putamen and the right Cd to the left STG and the ACC, respectively. Thus, the BG are not only involved in positively shaping the production of affective vocalization, but they might also inhibit certain functional processes in the neural network (Péron et al., 2013). The negative coupling of the left putamen with the left STG might help to filter unnecessary auditory feedback processed by the left auditory cortex, while focusing attention on vocal feedback processed in the right auditory cortex. Several studies have suggested that the left auditory cortex is especially sensitive to auditory information with high temporal resolution, while the right auditory cortex is mainly sensitive to spectral information (Zatorre and Belin, 2001), such as vocal pitch. Vocal pitch rather than the fine-grained temporal timing is an important cue to affective vocalizations, and thus its feedback processing is of high importance. This might be accompanied by a down-regulation of left-hemisphere mediated feedback on exact vocal timing (Lu et al., 2010; Chang and Zhu, 2013) and propositional speech processing (Ravizza et al., 2011) during affective prosody production as indicated by a negative left pSTG and left Put coupling. Besides the negative Put-STG coupling, we also found a negative coupling of the right Cd with the ACC. This might be a counter-regulation loop for the positive ACC-Put coupling. While the latter is necessary to release vocal patterns, the former might adaptively regulate this release especially under the condition of more controlled vocalizations. The present study involved controlled vocalizations, and this release regulation might thus suppress unintended vocal responses. Furthermore, it might online adapt the production in comparison of the memory stored representation for a fine-grained reproduction or imitation.

Our second main question concerned the functional role specifically of the IFG and the STG. These brain structures have been widely neglected in recent neural models of primate vocalizations (Jürgens, 2009; Lauterbach et al., 2013; Ackermann et al., 2014). The present data, however, indicated that both regions are an important part of the vocalization network. The STG subregions showed a close intra-hemispheric and bilateral coupling probably for the purpose of exchanging, monitoring, and online adjusting important acoustic information during vocal productions (Steinmann and Mulert, 2012; Parkinson et al., 2013; Kort et al., 2014). A more extended left-right STG coupling was found for the condition of repeating affective prosody, which involves both higher vocal control demands as well as short-term memory storage of vocal patterns. For the condition of repeating affective prosody we furthermore found a connection between right STG and right IFG. This points to the coupling of vocal monitoring accomplished by the IFG and auditory feedback processing in the STG (Tourville et al., 2008; Golfinopoulos et al., 2011). This coupling might be especially relevant during increased vocalization demands to repeat a vocalization accurately. Finally, the right STG regions also showed a functional connectivity to bilateral MC for vocal tract movements. Thus, motor commands to the vocal tract during affective vocalizations are directly influenced by right auditory regions (Greenlee et al., 2004; Frühholz et al., 2014b).

Besides the right STG, the bilateral MC showed also functional connections to right IFG. As mentioned before, the IFG is supposed to monitor the vocal output especially in terms of its paralinguistic and prosodic features, and might allow online corrections for unintended vocal behavior (Frühholz et al., 2014b). Thus, bilateral MC seems to be influenced mainly by a right lateralized network of brain regions involved in auditory feedback processing and vocal monitoring. This observation of a right lateralized network is related to our third major question outlined in the introduction. It supports results from auditory feedback processing pointing especially to right hemispheric regions involved in pitch control (Toyomura et al., 2007). The right lateralization is furthermore corroborated by our finding that the left IFG mainly shows negative functional connections with the left HC, but also with the left MC. The left rather than the right IFG is mainly involved in preparing and monitoring the linguistic dimension of vocal utterances, such as in speech (Blank et al., 2002). This left lateralized linguistic monitoring seems to be inhibited during the production of affective prosody, while the paralinguistic monitoring in the right hemisphere is increased. This is in accordance with the above discussed negative connectivity between the Put and the left pSTG. These findings support the general view of a dominant role of right frontal regions in affective prosody production (Ross and Monnot, 2008). They thus represent the first clear finding from a neuroimaging study pointing to right hemispheric predominance of the neural network underlying affective vocalizations.

Taken together, our data provide several new findings and important features about the neural network underlying the production of vocal emotions. First, compared to recent neural network models (Jürgens, 2009; Lauterbach et al., 2013; Ackermann et al., 2014) we critically extended the functional roles of several brain regions (ACC, BG) beyond their roles that have been proposed in these recent neural network models. The ACC might be involved in more controlled human affective vocalizations, especially in the release of affective intonations imposed on speech. The BG might not only influence the temporal patterning of affective speech, but they might also regulate this vocal output by a balance of regulatory and inhibitory network mechanisms. Second, besides this description of the extended roles of the ACC and the BG, we furthermore also confirmed the importance of the auditory-frontal network (Frühholz et al., 2014b), which has been largely neglected in recent network models (Jürgens, 2009; Ackermann et al., 2014). This auditory-frontal (i.e., STG-IFG) network seems to have a much more central role in conjunction with higher monitoring and auditory feedback demands during human affective vocalizations. Finally, we found a dominant role of a right hemispheric network underlying the production of affective vocalizations, which is especially dominant during the higher demanding imitation of affective vocalizations than during the evoked vocal expression of emotions. This finding is in line with recent studies in patients showing that right hemispheric lesions more strongly impair the accurate expression of vocal affect (Ross and Monnot, 2008). Future studies thus might record fMRI data in patients with selective lesion or functional impairments in one of the major nodes in the neural network proposed here to reveal further evidence for the functional role of the regions in the neural vocalization network.

A final note concerns some of the limitations of our study. First, the present study only investigated the neural network underlying the production of vocal anger. While we here focused on the highly arousing nature of vocal anger to especially test the role of ACC in arousal regulation during affective vocalizations, future studies need to investigate affective vocalizations of different valences to assess the generalizability of our neural network data. Second, we only tested a rather small number of human participants, and future studies might aim to test larger samples of participants. Yet, since we used a random-effects group analysis, that revealed significant results, our data seem to imply some generalizability. Third, movement related artifacts are one of the major sources of noise in fMRI experiments, and we cannot completely rule out some movement related induced activations in our study. We carefully checked head movements in our participants, however, and all participants moved less than half a voxel size in our study, which considerably minimizes the potential movement related artifacts. Fourth, the present study only involved rather standard scan settings with a common spatial resolution. Future studies might use high spatial resolution scanning to also more precisely determine signal in small brainstem structures that seem relevant to the neural vocalizations network, such as the PAG (Wattendorf et al., 2013). Finally, we might also have to mention some confounding factors in our study, that concern the state of high experimental control during the production of emotional prosody. Our study included affective vocalizations on command and participants were not in a natural emotional state during these vocalizations. Furthermore, participants produced vocalizations in a lying position in the scanner and were asked to restrain from head movements. This might also represent a rather unusual way of vocally expressing emotions. Future studies thus might investigate this neural network based on spontaneous vocalizations and based on real or induced emotional states.

## Conflict of Interest Statement

The authors declare that the research was conducted in the absence of any commercial or financial relationships that could be construed as a potential conflict of interest.

## Footnotes

^1^see http://vnl.psy.gla.ac.uk/

^2^http://afni.nimh.nih.gov/afni
